# Normative echocardiography data of myocardial adaptation to extrauterine life: a review of prospective studies

**DOI:** 10.3389/fped.2023.1192618

**Published:** 2023-06-16

**Authors:** Laura Mihaela Suciu, Irina Prelipcean, Amalia Făgărășan, Regan E. Giesinger, Patrick J. McNamara

**Affiliations:** ^1^ Department of Pediatrics, University of Medicine Pharmacy Science and Technology George Emil Palade of Targu Mures, Targu Mures, Romania; ^2^ Department of Neonatology, University of Rochester Medical Center Golisano Children’s Hospital at Strong, Rochester, NY, United States; ^3^ Department of Pediatrics, University of Iowa Stead Family Children’s Hospital, Iowa City, IA, United States

**Keywords:** transition, cardiocirculatory adaptation, ecocardiography, normal reference, newborns

## Abstract

Recent research has increased focus and interest in characterizing the physiology of the transition circulation using echocardiography. Critique of published normative neonatal echocardiography data among healthy term neonates has not been performed. We have performed a comprehensive literature review using the key terms: cardiac adaptation, hemodynamics, neonatal transition, term newborns. Studies were included if they had reported echocardiography indices of cardiovascular function in the presence of maternal diabetes, intrauterine growth restricted newborns and prematurity and had a comparison group of healthy term newborns within first seven postnatal days. Sixteen published studies evaluating transitional circulation in healthy newborns were included. There was marked heterogeneity in the methodologies used; specifically, inconsistency in time of evaluation and imaging techniques used makes it challenging to determine specific trends of expected physiologic changes. Some studies revealed nomograms for echocardiography indices, though limitations persist in terms of sample size, number of reported parameters and consistency of measurement technique. A comprehensive standardized echocardiography framework which includes consistent techniques for assessment dimensions, function, blood flow, pulmonary/systemic vascular resistance, and shunts pattern is warranted to ensure consistency in the use of echocardiography to guide care of healthy and sick newborns.

## Introduction

Transition to extrauterine life is one of the most challenging processes for the human body. Under normal conditions, the switch from the parallel fetal to serial neonatal circulation should happen immediately after birth triggered by lung aeration and umbilical cord clamping. *First*, a 10-fold increase in pulmonary blood flow (PBF) occurs, as pulmonary vascular resistance (PVR) decreases (1), augmenting left ventricle (LV) preload. *Second*, systemic vascular resistance (SVR) and arterial blood pressure (BP) rapidly increase by removing the connection between fetal and placental circulation. *Third*, the shunt at the patent ductus arteriosus (PDA) level switches from pure right-to-left to predominantly left-to-right further increasing PBF and LV preload. The vulnerability of transitional newborns relates to myocardial immaturity. Although the neonatal right ventricle (RV) is primed to work against a relatively high resistance circulation *in utero*, recent study suggest that RV appears to be more vulnerable among intrauterine growth restricted (IUGR) affected fetuses (2). In addition, impaired RV function is associated with adverse outcomes in newborns with neonatal encephalopathy undergoing therapeutic hypothermia (3). The LV myocardium, rich in non-compliant tissue, has a great vulnerability to increase his stroke volume for a given increase in afterload. Impaired myocardial performance is seen in many disorders of the neonatal period specifically, birth asphyxia, newborns of diabetic mother (IDM), sepsis, IUGR, and preterm birth. For example, fetuses compromised by *acute and severe* asphyxia are at risk of sustained elevation of PVR, low cardiac output (CO) secondary to impaired systolic performance (4) and low filling of the LV after birth (5). The adaptive response of the IUGR fetus, experiencing *chronic mild hypoxia* secondary to placental insufficiency, is characterized by redistribution of blood flow to the brain and heart (6). After birth, IUGR are at a great risk for hemodynamic instability due to low LV stroke volume (7), and higher systemic and PVR (8). The IDMs with cardiac hypertrophy are susceptible to decreased LV function and left outflow tract obstruction (9), increased PVR, and acute pulmonary hypertension (10). Further, their risk of cardiocirculatory instability is aggravated by greater myocardial vulnerability to coronary artery hypoperfusion (11).

## Rationale and purpose of the review

The primary aim was to review published literature related to echocardiography measurements of heart function, systemic and pulmonary hemodynamics, during the postnatal transition. Knowledge of the normal postnatal cardiovascular adaptive changes in healthy babies is vital to enable recognition of deviations from normal, and to aid understanding of mechanisms of disease and thresholds for intervention. Echocardiography is increasingly used for assessment of dysregulation of transitional physiology. The establishment of standardized imaging and measurement frameworks to characterize normative changes during the postnatal transition is essential to streamline future research efforts.

## Methods

Publications were identified from a systematic search of PubMed, conducted in November 2022. The search strategy included the following text terms: “transition”, “cardiocirculatory adaptation”, “echocardiography”, “normal reference values”, and “neonate”. The search was further refined by adding the terms' “systolic function”, “myocardial velocities”, and “functional indices”. The search time period was up to March 2023. The focus of the study was to characterize the cardiovascular transitional changes of the healthy term newborns.

Inclusion criteria for selecting studies were those that used echocardiography in the transitional period to describe: (i) longitudinal cardiovascular changes among healthy term newborns from low-risk pregnancies; (ii) comparative changes between healthy term versus preterm newborns; (iii) comparative changes between term IUGR or IDM patients and healthy term controls. Exclusion criteria were studies that focused on: (i) preterm newborns receiving respiratory support or treatment for PDA; (ii) newborns with congenital heart disease; (iii) newborns with arterial cord blood pH less than 7 or required admission to the neonatal intensive care unit; (iv) non-English language reports.

Two independent reviewers (LMS, IP) assessed all studies until consensus was reached. Four studies were excluded based on the aforementioned criteria leaving 16 studies for analyses, the oldest published in 1988 and the newest in 2018. Echocardiography indices were categorized as dimensional/structural, functional, resistance, flow, and shunt measurements. Details of the specific echocardiographic view [apical two-chamber (2C), four-chamber (4C), five chamber (5C), parasternal long axis, parasternal short axis, high parasternal, subcostal], measurement technique [M-mode, two-dimensional (2D), Color Doppler (CWD), pulse-wave Doppler (PWD), PWTD, tissue Doppler imaging (TDI), speckle tracking echocardiography (STE)], and compliance with American Society of Echocardiography recommendations. Additional variables included year of publication, record of investigators echo training, inter- and intra- observer variability data, sample size, and number of evaluation (time points).

## Results

Overall, 150 echocardiographic indices were reported in the selected studies. Specific cardiac parameters assessed in the selected reports included: RV and LV systolic and diastolic functional indices (10, 12–19); isolated RV dimensions and normative *z* scores (20); LV normative dimensional correlated with birth weight and functional indices (12); PVR indices (10, 13, 14, 19, 21); isolated LV systolic and diastolic function (22, 23); isolated RV systolic function (24); tricuspid annular peak systolic excursion (TAPSE) norms for birth weight and gestational age (25); RV and LV systolic function, mitral annular peak systolic excursion (MAPSE), TAPSE indexed to LV end-diastolic length (LVEDL) (26). A summary of study details including main findings, methods, strengths and limitations are presented in Table 1.

**Table 1:** Selected studies and investigators, key points and strength and limitations.

	Sample size	Echo-time points	Reported measurements	Key points	Strength	Limitation	Method
Jain et al., 2014 (20)	50 term	15 ± 2 h 35 ± 2 h	RV normative dimensions and functional indices (*Z* score)	PAAT fell, RVET unchanged, Septal flattening normalized. Most PDA's closed.	RV normative dimensional and functional indices 12–48 h. Three methods to investigate reproducibility	No evaluation <12 h No quantitative shunt assessment	2D, STE
Jain et al., 2016 (12)	50 term	15 ± 2 h 35 ± 2 h	LV dimensions and functional indices	LV dimensions correlated with BW. LV S’ lateral wall increased. Most PDA's closed.	LV normative dimensional and functional indices 12–48 h. Three methods to investigate reproducibility	No evaluation <12 h	2D, STE, TDI
Jain et al., 2018 (13)	15 term	<0.5, 2–3, 7–10 and 22–24 h	PVR, CO, Myocardial function Shunts	RV and LV-SV increased. PVR indices decline. LVO remained stable. All RV functional increased	Differential LV-RV adaptative response to loading conditions and PDA closure	Small sample size	2D, STE,
Noori et al., 2012 (14)	20 term	3–7, 9–14, 15–19 min and 24–48 h	(LVO, RVO SF) RV and LV function and flow	*Early transition*: Increased LVO but no changes in RVO. PDA changed to left-right. *Late transition*: LVO decreased and RVO increased	Serial echo data within first 20 min of life	Limited echo dataset.	2D
Negrine et al., 2012 (15)	16 term 27 preterm	15 h	LV and RV E/E’	Increased myocardial systolic and diastolic (E/E’) function increase with advancing GA. LV E/E’ was higher than RV E/E’ in both groups	Preterm population had lower diastolic velocities	No serial data. Preterm subjects on respiratory support.	2D, TDI
Clark et al., 2002 (24)	18 term 17 preterm	5, 26 and 49 h	RV volume, RVSV, RVO	RV volume decreased during normal transition. No change in RV-SV or RVO	No differences between term and preterm	No quantitative shunt assessment	2D
Evans et al., 1990 (21)	18 term 19 preterm	<12 h	TPV: RVET PDA shunts	Pulmonary artery pressure falls slower among stable preterms. PDA beyond day 3 is unusual in healthy preterms	Comparison of term vs. preterm infants	No serial data. No data on heart dimensions or function	2D, PW Color
Fouzas et al., 2014 (16)	30 healthy term and 30 term IUGR	Day 2 Day 5	LV dimensions and hemodynamics, RV and LV MPI	Different morphology, subclinical myocardial dysfunction, altered pattern of cardiovascular adaptation in IUGR	IUGR patients had evidence of cardiac dysfunction and distinct changes in transition.	No serial data of PBF, SBF hs-PDA excluded	2D, TDI
Ciccone et al., 2011 (17)	33 term 20 preterm	2.9 days	LV dimensions LV/RV systolic and diastolic function	Different LV wall and IVS dimensions, low systolic and diastolic function among preterms	Comparative data on LV and RV basal diameter and ventricular performance	No PBF, SBF No serial data	M, 2D, TDI
Kozak-Barany et al., 2001 (22)	25 term 20 preterm	2–5 days 1 month	LV systolic and diastolic function, LV mass	Lower LV diastolic function among preterms which improved by 1 month. LV mass increased with advanced age in both groups.	Comparative term vs. preterm data	No PBF, SBF, no data within first 48 h.	M, 2D
Koestenberg et al., 2011 (25)	258 term and preterm	<48 h	TAPSE to evaluate RV systolic function	Reference values of TAPSE and Z Score. TAPSE increased in a linear way from 26th to 41st week GA. No differences male/female.	Large sample size. Use of TAPSE which has good reproducibility and high specificity for abnormal RV systolic function.	No serial data. Preterms <29 weeks received prophylaxis for PDA closure. No other markers of RV function.	M
Eriksen et al., 2014 (26)	45 term 53 preterm (31–35 weeks)	*Term*: Day 3 and 3–4 months *Preterm*: Day 3 and 4–10 weeks	MAPSE, TAPSE and S’ indexed at LVEDL	LV and RV systolic function impaired among preterms. Indices increased with advanced GA and accelerated with postnatal age.	Longitudinal data with term vs. near-term comparison.	No serial measurement Shunts not assessed. Heterogeneity of groups according to respiratory state.	M, PW TDI
Johanson et al, 1988 (18)	10 term 21 preterm (28–35 weeks)	<4, 6–10, 12–16, and 25–34 days	TV and MV velocities	LV and RV diastolic function not dependent on GA	Reference value of diastolic filling in preterm and term	Small mixed sample. No data within first 30 h.	PW Doppler
Schierz et al., 2017 (19)	68 term 68 term IDM	24, 48 and 72 h	LV dimensions, LV EF/SF, MV & TV E, A, E/A, Tei index	Impairment LV systolic and diastolic among IDMs. Higher PAPs among IDM at 48 h	Impaired LV/RV diastolic & LV systolic disfunction, higher PAPs at 48 h in IDM patients.	No serial data reported. PDA and PFO not assessed	M, 2D, CW, PD
Katheria et al., 2012 (10)	18 term 32 term IDM	12, 24, 48 and 72 h in IDM. 12 and 48 h in term controls.	RVO, LVO and SVC flow. PFO and PDA.	LVO decreases and RVO increases over time in both groups. Delayed transition in IDM patients [More bidirectional PDA at 24 h but PAAT: RVET comparable between groups]	Longitudinal comparison of healthy term vs. IDM term patients.	IDM population limited to patients with good prenatal care (Thickness of the IVS not different among groups). Small sample size	2D, Doppler
Mori et al., 2004 (23)	396 patients	Single evaluation at mixed times (10 h, 4 days, child)	RV, IVS, LV Sw, Ew, Aw	LV and RV E: Ew ratio higher in neonates compared to children. RV relaxation may be impaired in the early neonatal period.	Large sample at variable time-points	Mixed data set.	PWDT

A, peak atrial flow velocity; Aw, peak early atrial systolic motion velocity; BW, birth weight; CO, cardiac output; E, peak early diastolic flow velocity; Ew, peak early diastolic motion velocity; GA, gestational age; hsPDA, hemodynamically significant patent ductus arteriosus; IDM, infants of diabetic mothers; IUGR, intrauterine growth restricted; IVS, interventricular septum; LV, left ventricle; LVEDL, left ventricular end diastolic length; LVO, left ventricle output; LV-SV, left ventricle stroke volume; MAPSA, mitral annular plane systolic excursion; MPI, myocardial performance index; MV, mitral valve; PAAT, pulmonary artery acceleration time; PAPs, pulmonary artery pressure systolic; PBF, pulmonary blood flow; PDA, patent ductus arteriosus; PVR, pulmonary vascular resistance; PW, pulsed wave; PWDT, pulsed wave doppler tissue; RV, right ventricle; RVET, right ventricle ejection time; RVO, right ventricle output; RV-SV, right ventricle stroke volume; SBF, systemic blood flow; SF, shortening fraction; STE, speckle-tracking echocardiography; SVC, superior vena cava; Sw, peak systolic motion velocity; TAPSE, tricuspid annular plane systolic excursion; TDI, tissue doppler imaging; TPV, time to peak velocity; TV, tricuspid valve.

The most commonly reported conventional echocardiography measurements, which are used in clinical practice, were TAPSE, mitral valve inflow (MV) E/A, shortening fraction (SF), ejection fraction (EF) by Simpson's biplane method, and ventricular outflow (VO) for the right and left ventricle. Additional measurements reported, but less commonly used in routine clinical practice, included TV E, TV A, TV E/A, RV S', RV E/E', and MV E, MV A, LV E/e', LV S' of lateral wall and interventricular septum, and LV stroke volume (SV) Table 2. Time of echocardiography evaluation varied among the publications; specifically, four studies reported measurements only in the first 24 h (13–15, 21), one study at 48 h only (25), six studies provided serial evaluation (<120 h) between early and late transition (10–12, 17, 19, 20, 24) and four studies compared measurements of neonates in early and/or late transition to older children (18, 22, 23, 26). The frequency of echocardiography assessment also was variable: single (*n* = 4) time-point evaluation (15, 17, 22, 23); paired (*n* = 5) time-points (12, 16, 20, 22, 26), three (*n* = 2) time-points (19, 24) or four (*n* = 4) time-points (10, 13, 14, 18). More than half of selected studies reported inter- and intra-observer variability rates (10, 12, 13, 16, 20–23, 25), while one quarter were performed by a single sonographer to minimize operator dependency error (14, 15, 24, 26). The sample size of healthy term newborns varied from ≤20 (13–15, 18, 21, 24) to ≥50 cases (12, 19, 20, 23, 25). Echocardiography methods included conventional measurements only in eight studies (10, 14, 18, 19, 21, 22, 24, 25), conventional and color/tissue Doppler echocardiography (cTDI) in five studies (15–17, 23, 26) and advanced myocardial evaluation using STE in three studies (12, 13, 20).

**Table 2:** Relevance according to the frequency of echo measurement in the selected studies.

	Reported parameter	Study^a^	Range limits
Right ventricle	TAPSE	(18, 20, 25, 26)	*Z* score calculator for GA
	TV A	(15–20, 23)	48.9 cm/sec in preterm to 54.9 cm/sec in term infants
	TV E	(15–20, 23)	38.2 cm/sec in preterm to 47.9 cm/sec in term infants
	TV E/A	(13, 15–20, 23)	0.78 in preterm to 0.88 in term infants
	RV E/e’	(15, 16, 20)	6.11 ± 1.43
	RVO (within first 24 h)	(10, 13, 14, 24)	235–255 ± 0.3 ml/kg/min
	S’ -RV	(15–17, 20, 23, 26)	6.2–6.6 cm/sec
Pulmonary vascular resistance	PAAT, RVET, PAAT: RVET	(13, 21)	PAAT = 33–35 ms RVET = 166–216 ms PAAT: RVET = 5.1–4 PAPs = 12 mmHg
	Shunt at the PDA level	(14)	
	Estimated pulmonary artery pressure from TR jet (within first 24 h)	(10, 19)	
	Interventricular septal flattening	(20)	
Left ventricle	MV A	(12, 15–19, 22, 23)	43.2 cm/sec in preterm to 47 cm/sec in term infants
	MV E	(12, 15–19, 22, 23)	32.7 cm/sec in preterm to 61.5 cm/sec in term infants
	MV E/A	(12, 15–19, 22, 23)	<1 in preterm to ≤1 in term infants
	LV E/e’	(15–17)	8.31 ± 1.67
	LVO (within first 24 h of age)	(10, 12–14, 16)	138–143 ml/kg/min
	LVSV	(13, 14, 24)	3.3–4.2 ml/kg/min in the first 24 h
	EF Simpson	(12, 13, 16, 17, 19, 24)	55%–65%
	SF	(12, 14, 16, 17, 19, 22, 23)	28%–40%
	S’-LV lateral wall	(12, 13, 15–17, 23, 26)	5.3–5.5 cm/sec
	S’-interventricular septum	(12, 17, 23, 26)	3.7–3.9 cm/sec

TAPSE, tricuspid annular plane systolic excursion; TV, tricuspid valve; A, peak atrial velocity; E, peak early velocity; RV, right ventricle; E, peak early diastolic flow velocity; e’, peak early diastolic motion velocity; RVO, right ventricle output; S’, peak systolic motion velocity; PAAT, pulmonary artery acceleration time; RVET, right ventricle ejection time; PDA, patent ductus arteriosus; MV, mitral valve; LV, left ventricle; LVO, left ventricle output; SV, stroke volume; EF, ejection fraction; SF, shortening fraction.

^a^ Number refers to paper listed in References.

### Dimensions and structure

Echocardiography dimensions were adjusted for birth weight, sex, and gestational age (12), indexed for body surface area (22), or adjusted for heart size (i.e., LV end-diastolic length) (26). LV linear dimensions were obtained from apical 4Ch, long or short axis parasternal view or both, either using M-mode (17, 22), by joining straight lines between basal segments of the LV walls (20), or as the distance from the apical epicardium to the level of the septal attachment of the mitral valve (26). LV dimensions were measured at end-diastole (20), or at both end-diastole and end-systole (17); of note, some studies do not report the specific phase of acquisition in the cardiac cycle (26). Most RV linear dimensions were obtained from multiple views; specifically, the RV focused apical 4-chamber view (20, 24) or parasternal long axis (20), or RV focused 3-chamber view (13) were used most. Overall, among healthy term newborns, LV dimensions remain unchanged and RV volume decreased during the transition; of note, a positive linear relationship between newborns' weight and longitudinal systolic function (TAPSE and MAPSE) was found (20, 26). Among selected studies, IUGR newborns were found to have more dilated LV and interventricular septum hypertrophy (16) and IDM were characterized by LV stiffness and myocardial hypertrophy (19).

### Heart function

**Figure 1:**
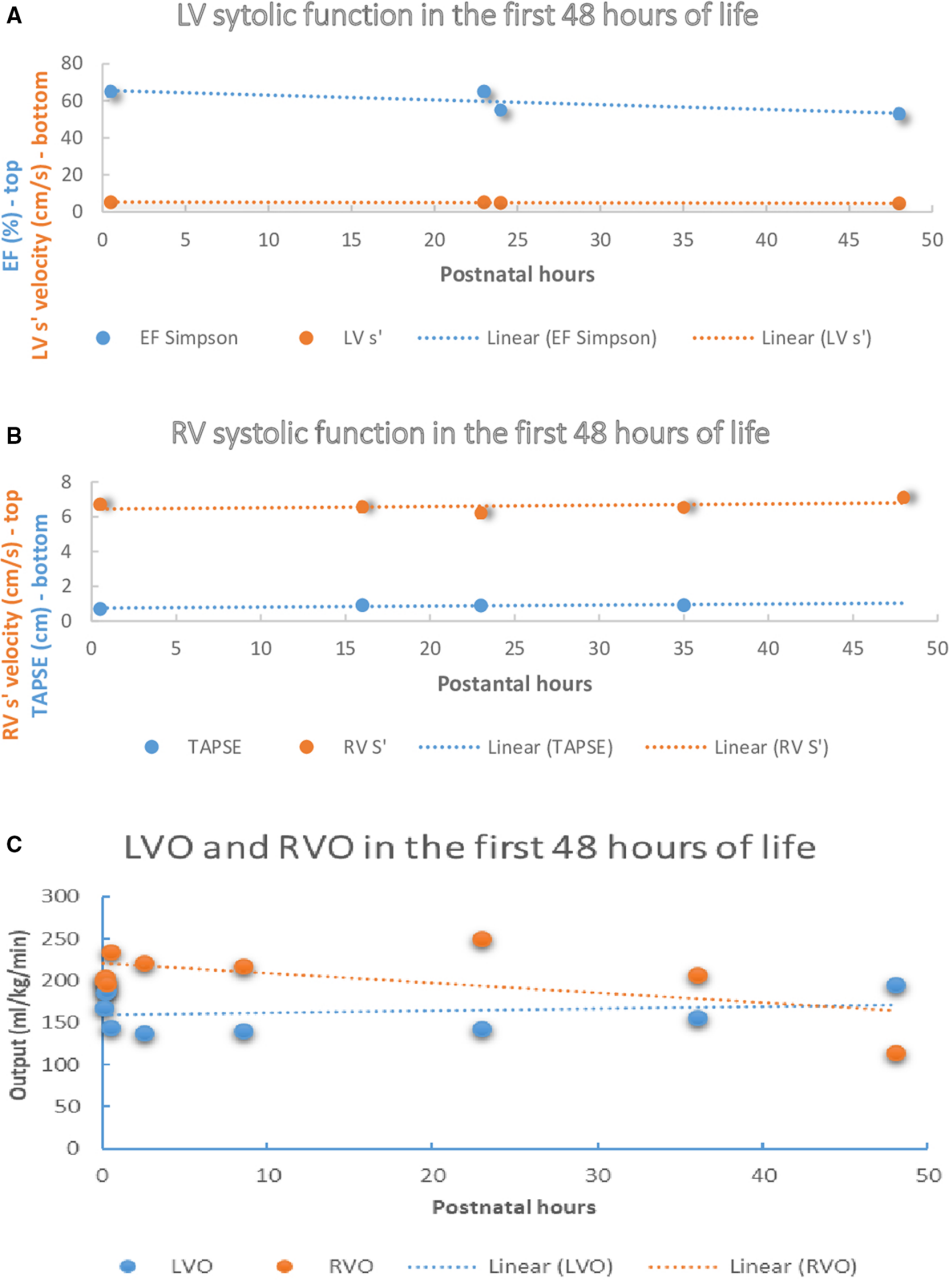
Left and right ventricle systolic function and output in the first 48 h of life amongst healthy term newborns. (**A**) LV systolic function in the first 48 h of life among healthy term newborns in selected studies (12, 13). (**B**) RV systolic function in the first 48 h of life among healthy term newborns in selected studies (13, 16, 20). (**C**) RVO and LVO in the first 48 h of life among healthy term newborns in selected studies (12–14, 16, 24). LV, left ventricle, LV S’, peak systolic motion velocity, EF, ejection fraction, TAPSE, tricuspid annular plane systolic excursion, RV, right ventricle, RV-S’, Peak systolic motion velocity, LVO, left ventricle output, RVO, right ventricle output.

Ejection fraction using Simpson biplane method and shortening fraction from the long axis parasternal view were the most frequently reported indices of LV systolic performance. Overall, conventional indices of LV systolic performance were unchanged during the transition; however, a marginal increase in indices of LV diastolic function (mitral E/A or systole': diastole' ratios >1.0) was noted during the first 48 postnatal hours. Studies using TDI showed that LV myocardial velocities were unchanged during first 48 h (Figure 1A); however, marginal reductions in LV lateral wall s' were noted. Some authors reported higher RV fractional area change measured from RV-3C than RV-4C (20) views, which has prompted use of global FAC. Studies using TDI demonstrated higher RV myocardial velocities compared to LV (15), and higher trans-tricuspid velocities increase with advancing chronological age, but E/A ratios were unchanged. A positive linear relationship between RV myocardial velocities (RV-S') and longitudinal systolic function (TAPSE) with the advancing postnatal age was found among term newborns during physiological transition (Figure 1B).

### Flow

Some authors report the velocity time integral (VTI), which is a trace of the pulse wave Doppler waveform acquired at the level of the hinge point of the aortic or pulmonary valves. LV-VTI and RV-VTI are most frequently performed in apical 5C and PLAX views respectively. Although mean LVO evaluated at 4 different time points within first 24 h after birth remained unchanged, RVO increased during the same period. In addition, the RVO: LVO ratio was >1.5 reflecting ongoing RV dominance after birth amongst healthy term newborns (13, 20). After the first 72 h RVO and LVO values were comparable among studies reporting these indices during the late transition (14, 16, 24) (Figure 1C). Some authors used regression analyses to test the effect of the heart rate on the selected parameters (26), whereas other studies used linear regression to assess relationship between LVO and RVO with cerebral blood flow and cerebral regional saturations (14), or to evaluate the relation of the LVO, RVO and superior vena caval (SVC) flow with infant characteristics (10).

### Resistance

Serial evaluation of pulmonary arterial pressure and PVR were performed using indirect measures [pulmonary artery acceleration time (PAAT), right ventricular ejection time (RVET), PAATi (indexed to total cardiac cycle duration) and indexed PVR (13, 21), interventricular septal flattening (20), direction of PDA flow (21), or estimated pulmonary pressure from tricuspid regurgitation (TR) jet (10, 19)]. The mean value of PAAT and RVET, measured at 4 different time points, increased while PVRi ratio (RVET: PAAT) decreased term newborns within 24 h of birth (13). PAAT and RVET are time dependent indices and, therefore, influenced by heart rate; however, changes in both indices were detected during first 24 h.

### Shunts

The most common view for PDA interrogation was the high parasternal view, and flow was evaluated by PWD, CWD, or according to the pulmonary artery flow pattern (22). The PDA shunt became predominantly left-to-right within minutes of birth and closed or small with restrictive flow by 24–48 h in 100% cases (12, 13, 20, 21). In addition, a persistent bidirectional PDA shunt beyond 10–15 h was considered “atypical” (13). Atrial level shunts were exclusively left-to-right in healthy term neonates, irrespective of chronological age (20), with higher incidence of atrial level shunts as chronological age advances (12).

## Discussion

Aside from known physiological concepts of neonatal transition to extrauterine life, it is challenging to determine any novel trends and refine our knowledge of early physiologic changes in newborns due to the lack of normative data. Although notable, the available literature remains limited due to small sample size (20–50 newborns) and inconsistent study design. Currently only 4 studies have reported longitudinal cardio circulatory adaptation to extra uterine life among healthy term neonates (12–14, 20). Most studies have focused on characterizing differences in adaptative cardiovascular mechanisms between term and preterm myocardium. Timing and frequency of echocardiography evaluation also varied among publications, with some studies reporting isolated measurements in the first 24–48 h, while others reported serial data between early and late transition. More than half of selected studies reported inter and intra variability rates, while few had images performed by a single sonographer to minimize operator dependency error (14, 15, 24, 26).

Some variability in imaging protocols is expected, given the lack of unified training programs, but the magnitude of differences in current reports are substantial. Most studies (*n* = 8) included only conventional echocardiography measurements, with 5 studies providing color tissue Doppler imaging (cTDI) and 3 reporting STE derived measurements. While some studies were designed to provide reference range and *z* score for certain parameters for diagnostic/predictive value (12, 20, 25), there are insufficient data to produce true normative data. Sample size is sufficiently small that it is not possible to appraise the influence of racial, sex or ethnicity on normative datasets. None of the selected studies report the prognostic value of the echocardiography parameters on short- or long-term outcomes.

Consistency, in how images are acquired, and measurements are performed, is essential when interpreting echocardiography data. Half of studies were performed by neonatologists (10, 12–15, 19, 20, 24) with the remaining by cardiologists (16–18, 21–23, 25, 26). Little information was provided on sonographer credentials or training, nor the steps taken to minimize operator-dependent variability. The most commonly reported measurements were LV and RV output, the presence of the PDA level shunts, TAPSE, PAAT and RVET, tricuspid and mitral inflow, and RV systolic myocardial velocity (RV-S'). Importantly, due to persistence of fetal channels in the transitional period, many of the reported measurements are influenced by flow; however, the confounding effects of shunts and flow patterns/direction were not reported among some studies (18, 19, 26) while others excluded newborns with hemodynamically significant PDA (16, 22, 25). In addition, specific measurement techniques were inconsistently reported making it difficult to combine datasets.

As highlighted previously, the postnatal period is characterized by important physiologic changes which are likely to have a major impact of cardiovascular adaptation. Unfortunately, few studies have performed longitudinal evaluation which makes interpretation of hemodynamic datasets in the context of disease states difficult. Where data exists, it is interesting to note that in the immediate transitional period (<30 min) left ventricular output increases, likely due to a combination of increased heart rate, changes in PDA flow, and increased LV preload (Figure 1C). Immediate changes in LV function after birth could be explained by the relative increase in LV preload after birth due to an increase in left to right shunt at the level of FO, increased LV afterload after elimination of the placental circulation, high likelihood of early PDA closure, and augmented postnatal diuresis. One study revealed that RV myocardial velocities were greater than LV (15) and concluded it aligned with the concept of persistence of RV dominance in the early postnatal period; however, this can also be explained by the mechanics of the LV contraction that is only partially affected by the longitudinal shortening (27). Notably, the compiled results in this review cannot be extrapolated to newborns of diabetic mothers, growth restricted newborns, or other populations with abnormal cardiovascular adaptations.

We acknowledge several limitations of this present analysis. *First*, only reports written in English were included. Therefore, normative data in these studies may not be globally applicable due to racial or ethnicity differences. *Second*, we are unable to obtain information about the specific training of individuals performing the echocardiography studies. By recognizing this limitation, we aim to promote transparency and encourage documentation of the expertise for the clinicians involved. *Third*, the lack of available information regarding delayed cord clamping could have potentially influenced the reported measurements since healthy term neonates with delayed cord clamping are reported to undergo a more physiological transition (28). *Fourth*, there remain gaps in the provision of normative data for some measurements. For example, there are little data on indices of LV afterload including systemic vascular resistance. In addition, some time-dependent indices like the isovolumic relaxation time would benefit from indexing to heart rate or some other comparable time-dependent index. *Fifth*, it is not clear how often these measurements are used in routine clinical practice; in particular, usage patterns for novel measurements such as PAAT, RVET, or advanced indices of heart function, in guiding treatment is an important gap in the literature. Finally, we were unable to verify the quality of imaging studies and steps taken to ensure consistency in measurement techniques between studies.

## Conclusions

This review of the neonatal transition literature highlights major gaps in normative echocardiography data. There remains a great need for standardized echocardiography measurement techniques, protocols, and training to advance our ability to recognize and intervene in the care of a baby with an abnormal transition. Thus, we propose the need to involve relevant stakeholders and develop standardized echocardiography framework for any future research in this domain, with consistent time-points for image acquisition [e.g., immediate (first hour), early (1–24 h), and late transition (48–72 h)], agreed standards for both imaging and measurement techniques, and which target diverse perspectives to ensure racial and ethnic variance is considered.
